# Molecular Research on Amyloidosis

**DOI:** 10.3390/ijms24086980

**Published:** 2023-04-10

**Authors:** Kazufumi Nakamura

**Affiliations:** Department of Cardiovascular Medicine, Graduate School of Medicine, Dentistry and Pharmaceutical Sciences, Okayama University, Okayama 700-8558, Japan; ichibun@cc.okayama-u.ac.jp; Tel.: +81-86-235-7351; Fax: +81-86-235-7353

Amyloidosis is a large group of diseases that are caused by the deposition of insoluble amyloid fibrils formed by misfolded soluble proteins in organs or tissues. The most common types of systemic amyloidosis are amyloid light-chain (AL) amyloidosis, amyloid A (AA) amyloidosis, and transthyretin (TTR) amyloidosis, which are caused by the deposition of amyloid fibrils constituted from immunoglobulin free light chains (FLCs), serum amyloid A protein, and TTR, respectively. Although our understanding of amyloidosis pathobiology and therapy has increased substantially in recent years, molecular mechanisms, molecular therapeutic targets, and molecular imaging have not been fully elucidated.

This Special Issue of the International Journal of Molecular Sciences, entitled “Molecular Research on Amyloidosis”, contains a total of seven submissions. Three original papers and four review articles provide new information on the mechanisms, diagnosis, and treatment of amyloidosis.

Saito et al. [1] reviewed recent advances in the mechanisms and treatment of cardiac amyloidosis. Cardiac involvement is a major prognostic factor in patients with systemic amyloidosis. For ATTR and AL amyloidosis, which exhibit cardiac involvement, therapies that inhibit the production of the causative protein have been developed and have improved the prognosis. With regard to the treatment of ATTR, studies have been conducted on the disruption of TTR aggregation, the stabilization of TTR tetramers, the inhibition of TTR synthesis, the removal of TTR aggregates, and post-liver transplant anti-seeding therapy, in addition to some clinical applications that have been carried out. Tafamidis [2], which stabilizes TTR tetramers, and patisiran [3], which inhibits TTR synthesis, have been reported to be effective in patients with ATTR cardiac amyloidosis. However, a method for removing deposited amyloid has not been established. As for the treatment of AL, the elimination of light-chain sources, the disruption of light-chain aggregation, the removal of amyloid deposits, and the stabilization of amyloidogenic light chains have been studied, and some have been applied clinically. Daratumumab is a monoclonal antibody that binds to CD38, an antigen highly expressed on the surface of AL amyloidosis plasma cell clones. Daratumumab-based treatment has been reported to be effective in patients with AL cardiac amyloidosis [4].

Ikura et al. [5] reviewed recent topics on the molecular mechanisms of pathogenesis and therapeutic strategies for AL amyloidosis. To elucidate the pathogenesis of AL, many basic studies using human specimens have been performed, and genetic mutations associated with AL [6], the characteristics of amyloidogenic light chains (LCs) [7], and the structural specificity of amyloid fibrils [8,9] have been clarified. Furthermore, the mechanisms of cell and tissue damage, such as the mass effect of amyloid deposition and the toxicity of prefibril LCs, are being elucidated [10]. To date, many clinical studies have focused on therapeutic agents for the disease, especially chemotherapy. Since the rapid reduction of FLCs, the protein responsible for amyloid, is necessary to achieve hematologic effects, various anticancer agents targeting neoplastic plasma cells have been used in the treatment of this disease [4].

Identification of the type of amyloid is clinically essential, as prognosis and treatment strategies vary with specific amyloid diseases. Immunohistochemical staining of tissue biopsies is commonly used for amyloid typing, but newer technologies, such as immunoelectron microscopy and laser dissection mass spectrometry, appear superior to immunohistochemistry in identifying amyloid protein type [11]. Goldis et al. also reviewed the utility of the Western blotting analysis of proteins extracted from tissue biopsies [12]. In addition to immunohistochemical staining, noninvasive methods such as serological testing and imaging are used for diagnosis. Bone scintigraphy enables the diagnosis of cardiac ATTR amyloidosis in patients who do not have a monoclonal gammopathy [13]. Recent studies found that by using this method, the prevalence of cardiac ATTR amyloidosis in patients with heart failure with a preserved ejection fraction is 13–18% [14,15,16].

The mechanisms of bone tracer uptake into the heart are not yet fully understood. Mori et al. reported that calcified microparticles were found in endomyocardial biopsy samples from ATTR-CA patients, but no elevated expression of bone metabolism-related genes was observed, suggesting that active calcification-promoting mechanisms were not involved [17]. The mechanisms explaining the accumulation of bone tracers in the hearts of ATTR-CA patients require further investigation.

Apolipoprotein A-I (ApoA-I) amyloidosis is a rare protein misfolding disease in which fibrils of the N-terminal domain of the protein accumulate in several organs and cause damage. Giudice et al. reported that ApoA-I variants exert cytotoxicity in a time-specific and cell-type-specific manner, possibly due to protein accumulation in lysosomes [18], and that the autophagic process is inhibited in the presence of the L75P-ApoA-I amyloidogenic variant in stably transfected human hepatocyte carcinoma cells [19].

None of the papers in this Special Issue proposed new translational works or clinical randomized trials. It is hoped that academic research and industry will work together in the future to promote further translational works and clinical trials.

## Abbreviations

AA	amyloid A
AL	amyloid light chain
ApoA-I	Apolipoprotein A-I
ATTR	transthyretin amyloidosis
ATTR-CA	transthyretin cardiac amyloidosis
FLCs	free light chains
LCs	light chains
TTR	transthyretin
